# Symptoms and syndromes associated with SARS-CoV-2 infection and severity in pregnant women from two community cohorts

**DOI:** 10.1038/s41598-021-86452-3

**Published:** 2021-03-25

**Authors:** Erika Molteni, Christina M. Astley, Wenjie Ma, Carole H. Sudre, Laura A. Magee, Benjamin Murray, Tove Fall, Maria F. Gomez, Neli Tsereteli, Paul W. Franks, John S. Brownstein, Richard Davies, Jonathan Wolf, Tim D. Spector, Sebastien Ourselin, Claire J. Steves, Andrew T. Chan, Marc Modat

**Affiliations:** 1 School of Biomedical Engineering and Imaging Sciences, King’s College London, 9th floor, Becket House, 1 Lambeth Palace Road, London, SE1 7EU UK; 2 Boston Children’s Hospital and Harvard Medical School, Boston, MA USA; 3 Clinical and Translational Epidemiology Unit, Massachusetts General Hospital, Boston, MA USA; 4 Department of Women and Children’s Health, School of Life Course Sciences and the Institute of Women and Children’s Health, King’s College London, London, UK; 5 Department of Medical Sciences and Science for Life Laboratory, Uppsala University, Uppsala, Sweden; 6 Department of Clinical Sciences, Lund University Diabetes Centre, Jan Waldenströms gata 35, 21428 Malmö, Sweden; 7 Zoe Global Limited, London, UK; 8 Department of Twin Research and Genetic Epidemiology, King’s College London, London, UK

**Keywords:** Epidemiology, Comorbidities, Respiratory signs and symptoms

## Abstract

We tested whether pregnant and non-pregnant women differ in COVID-19 symptom profile and severity, and we extended previous investigations on hospitalized pregnant women to those who did not require hospitalization. Two female community-based cohorts (18–44 years) provided longitudinal (smartphone application, N = 1,170,315, n = 79 pregnant tested positive) and cross-sectional (web-based survey, N = 1,344,966, n = 134 pregnant tested positive) data, prospectively collected through self-participatory citizen surveillance in UK, Sweden and USA. Pregnant and non-pregnant were compared for frequencies of events, including SARS-CoV-2 testing, symptoms and hospitalization rates. Multivariable regression was used to investigate symptoms severity and comorbidity effects. Pregnant and non-pregnant women positive for SARS-CoV-2 infection were not different in syndromic severity, except for gastrointestinal symptoms. Pregnant were more likely to have received testing, despite reporting fewer symptoms. Pre-existing lung disease was most closely associated with syndromic severity in pregnant hospitalized. Heart and kidney diseases and diabetes increased risk. The most frequent symptoms among non-hospitalized women were anosmia [63% pregnant, 92% non-pregnant] and headache [72%, 62%]. Cardiopulmonary symptoms, including persistent cough [80%] and chest pain [73%], were more frequent among pregnant who were hospitalized. Consistent with observations in non-pregnant populations, lung disease and diabetes were associated with increased risk of more severe SARS-CoV-2 infection during pregnancy.

## Introduction

The COVID-19 pandemic is caused by the SARS-CoV-2, a newly identified enveloped RNA-β-coronavirus^1,2^. Early on, pregnant women were regarded as vulnerable group, at greater risk of severe morbidity and mortality, based on previous studies of smaller coronavirus outbreaks, and in consideration of the theoretical risks associated with immunosuppression of pregnancy^3–5^. However, substantial literature has now documented that, among hospitalized pregnant women, antecedent symptoms and risk factors for severe disease are similar to those outside pregnancy^6^. In addition, few hospitalized pregnant women require admission to intensive care or intubation, although preterm birth, Caesarean delivery, and stillbirth may be increased compared with women without COVID-19, and vertical transmission is possible (86 studies to 8 Jun 2020)^7–10^. SARS-CoV-2 positive patients develop dry cough, fever, dyspnea, fatigue and bilateral lung infiltrates on imaging in the severe cases^11^. Hospitalized pregnant women positive for SARS-CoV-2 manifest similar symptoms^7,12,13^. However, little is known about pregnant women affected by SARS-CoV-2 infection in the community, many of whom recover without hospitalization^14^.

Smartphone and web-based applications for population-based syndromic surveillance are citizen science tools that can facilitate rapid acquisition of extensive epidemiological data as a pandemic evolves^15^. These data can inform public-health policies, enhance the speed of the healthcare response, shape the community services, and alert the general population to urgent health threats^16^. Smartphone applications (apps) were used prior to the COVID-19 pandemic to remotely advise on prenatal health, and maternal health behaviours, including gestational weight gain and smoking cessation^17^. Many eHealth initiatives were launched at the onset of the pandemic, with most using single, cross-sectional reporting methods to inform SARS-CoV-2 epidemiology^18^. We present findings from a unique, longitudinal community-based symptom-tracking system that identified both test positive and suspected (but untested) SARS-CoV-2 infected pregnant women, who were followed prospectively to assess the need for hospitalization. Furthermore, we replicated key findings, using an independent, cross-sectional symptom survey.

We present data from a cohort of women of childbearing age, including pregnant women who reported test-positive to SARS-CoV-2. Despite presenting a wide spectrum of disease manifestations, these pregnant women rarely required hospitalization.

In order to include non-tested subjects who developed symptoms during the onset of the pandemic, when testing resources were still limited, we developed a model to predict positivity to SARS-CoV-2 based on symptoms, specific to female population in childbearing age. We sought to characterize the differences in the SARS-CoV-2 symptom profiles and severity between pregnant and non-pregnant women who did and did not receive hospitalization. We also identified demographic characteristics and comorbidities that modified symptom severity of SARS-CoV-2 in pregnancy.

## Results

We developed a symptom-based prediction method to identify suspected COVID-19 cases among women aged 18 to 44 years from a discovery cohort. Results were replicated in an independent, cross-sectional cohort with different survey methodology.

### Cohort characteristics and COVID-19 outcomes

The discovery cohort (N = 400,750 participants) was obtained from women (aged 18–44) in the test subset only. It includes longitudinal records from 14,049 pregnant and 386,701 non-pregnant women who had a median duration of follow-up of 18 days (IQR [6–34]) and contributed to an average of 6.6 reports per woman. Among the 45% of pregnant women who self-reported their gestation week at baseline, 14% were in the first trimester, 43% were in the second trimester, and 43% were in the third trimester. The replication cohort consisted of N = 1,344,966 cross-sectional surveys from women aged 18–44. One-time surveys were administered over the 9 week period, at average rate of about 149 thousand surveys per week, using survey methodology. There were 41,796 surveys from women who indicated they were pregnant (3.1% of the source population). Demography was consistent with US age-specific pregnancy rates and stable over the survey period^19^.

Demographic details are shown in Table 1, together with testing rates. In the discovery cohort, we identified 629 and 25,061 pregnant and non-pregnant women, respectively, who were suspected positive for SARS-CoV-2 infection based on the symptom-score-based imputation method. Of these suspected positive, 21 (3.3%) pregnant and 591 (2.4%) non-pregnant were hospitalized, respectively. In the replication cohort, the proportion of 1,076 (2.9%) suspected positive pregnant was slightly lower compared to 44,772 (4.0%) suspected positive non-pregnant.

**Table 1:** Characteristics of the two cohorts, presented as percentages and means (standard deviations) in the cohorts.

	Discovery cohort			Replication cohort		
	All (N = 400,750)	Non-pregnant (N = 386,701)	Pregnant (N = 14,049)	All (N = 1,344,966)	Non-pregnant (N = 1,303,170)	Pregnant (N = 41,796)
Age (years) (not age-standardized)	32.1 (7.2)	32.1 (7.3)	32.4 (4.9)	29.0 (0.02)	29.0 (0.01)	29.0 (0.05)
Tested	7.0%	6.1%	8.0%	2.5%	2.4%	2.7%
Positive	0.6%	0.7%	0.6%	0.4%	0.4%	0.4%
Negative	5.5%	4.9%	6.2%	2.2%	2.1%	2.2%
Suspected	5.6%	6.7%	4.5%	3.5%	4.0%	3.0%
**Comorbidities**						
Diabetes	1.8%	1.2%	2.3%	3.9%	3.5%	4.3%
Lung	12.9%	12.8%	11.3%	19.3%	19.8%	18.8%
Heart	0.6%	0.5%	0.6%	0.8%	0.9%	0.7%
Kidney	0.3%	0.4%	0.3%	0.6%	0.7%	0.5%
Cancer	0.1%	0.2%	0.1%	0.9%	1.1%	0.8%
Symptom severity	0.07 (0.11)	0.07 (0.11)	0.04 (0.09)	0.08 (0.0005)	0.08 (0.0003)	0.07 (0.001)
Test positive and hospitalized^a^	0.09%	0.07%	0.1%	0.06%	0.03%	0.09%
Suspected positive and Hospitalized^a^	0.16%	0.16%	0.15%	0.17%	0.12%	0.23%

Except for group age, percentages and means are age standardized to the pregnant population age distribution in each cohort. Adjustment for survey weights was applied to the replication cohort. Self-report of being seen at a hospital was used as a proxy for hospitalization in the replication cohort.

^a^Hospitalization not queried in replication cohort. Proportion of who tested positive or were suspected positive and who reported seeking care at a hospital for symptoms in the prior 24 h provided as a proxy.

**Figure 1:**
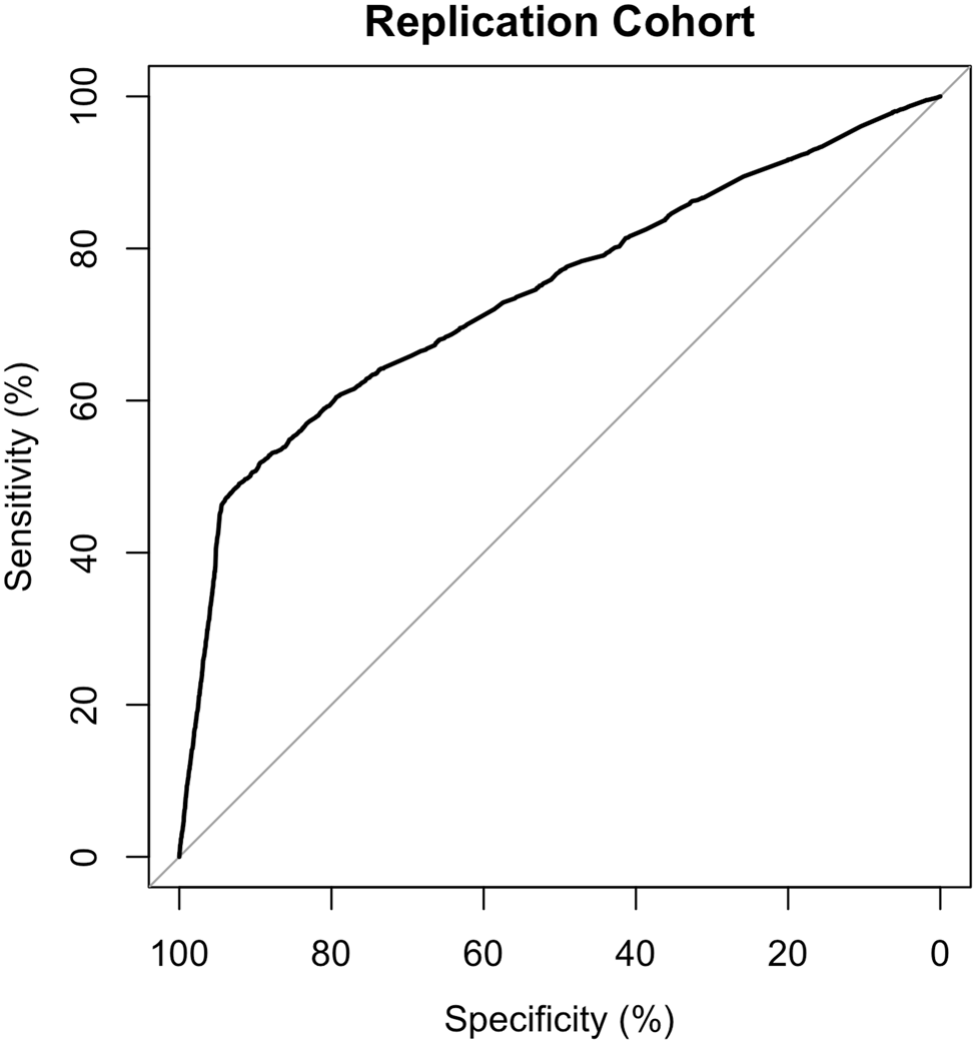
Receiver operating characteristics curve showing validation of the imputation of SARS-CoV-2 test status using the mapped symptom score probability in the replication cohort. Area under the curve is 74%.

Validation of the imputation method in a subset of the discovery cohort, and in the replication cohort is depicted in Fig. 1, with additional sensitivity analyses in Supplementary Information S2.

### Symptomatic, syndromic and severity predictors

**Figure 2:**
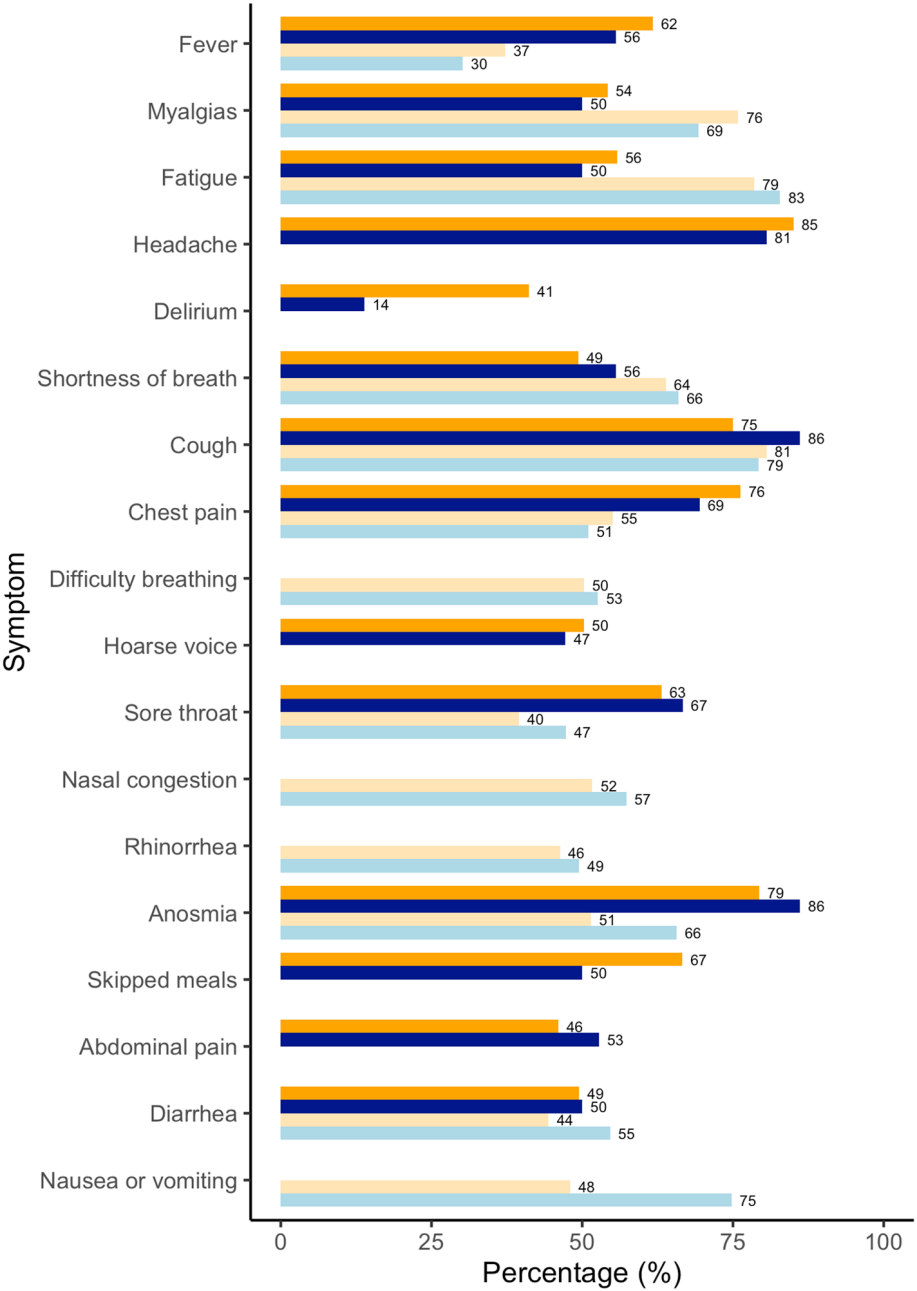
Comparison of symptoms presentation in the discovery (DC) and replication (RC) cohorts. Results refer to non-pregnant (orange) and pregnant (blue) women tested positive and suspected positive for SARS-CoV-2 and who required hospitalization (in DC, darker shade) or were seen at the hospital (RC, lighter shade). Results are reported as age-standardized percentage of women reporting each symptom in each sub-cohort.

Frequency of symptoms and body system clusters is reported in Table 2 for hospitalized women in the discovery cohort, and for women who reported being seen at a hospital for their illness in the replication cohort (as hospitalization data were not available). In the table, data are reported by pregnancy status and further subdivided into SARS-CoV-2 test positive or suspected COVID-19 status. Figure 2 graphically depicts the frequency of symptoms after grouping SARS-CoV-2 test positive and suspected COVID-19 cases, in the same two groups (pregnant, non-pregnant) and in the two cohorts. In the discovery cohort, the most frequent symptoms in the hospitalized pregnant women positive for SARS-CoV-2 were persistent cough, headache and anosmia (all 80.0%), chest pain (73.3%), sore throat and fatigue (66.7%). In the replication cohort, among pregnant test positive women who were seen at the hospital for their illness, the most frequent symptoms were fatigue (87.5%), cough (84.6%), nausea or vomiting (78.2%), muscle pain (76.2) and anosmia (75.2%).

**Table 2:** Frequencies and percentage values of presentation of each symptom among hospitalized in the discovery cohort, and among all women who self-reported being seen at a hospital for their illness in the replication cohort (as hospitalization data were not available).

Cluster (body system)	Symptom	Discovery cohort				Replication cohort			
		Hospitalised non pregnant positive (N = 229)	Hospitalised non pregnant suspected positive (N = 591)	Hospitalised pregnant positive (N = 15)	Hospitalised pregnant suspected positive (N = 21)	Seen at hospital, non-pregnant positive (N = 300)	Seen at hospital, non-pregnant suspected positive (N = 1395)	Seen at hospital, pregnant positive (N = 29)	Seen at hospital, pregnant suspected positive (N = 75)
Inflammation	Fever	151 (65.9)	359 (60.7)	8 (53.3)	12 (57.1)	135 (48.1)	514 (39.0)	12 (50.6)	19 (29.9)
	Unusual muscle pain	121 (52.8)	338 (57.2)	9 (60.0)	9 (42.9)	199 (69.0)	1,048 (77.0)	19 (76.2)	52 (71.8)
	Fatigue	125 (54.6)	345 (58.4)	10 (66.7)	8 (38.1)	207 (65.9)	1,142 (79.8)	24 (87.5)	61 (84.0)
Neurologic	Headache	185 (80.8)	516 (87.3)	12 (80.0)	17 (81.0)	NA	NA	NA	NA
	Delirium	88 (38.4)	253 (42.8)	4 (26.7)	1 (4.8)	NA	NA	NA	NA
Cardiopulmonary	Dyspnea	113 (49.3)	316 (53.5)	9 (60.0)	11 (52.4)	166 (54.8)	913(65.1)	20 (73.6)	47 (66.9)
	Persistent cough	178 (77.7)	438 (74.1)	12 (80.0)	19 (90.5)	202 (68.2)	1,161 (82.3)	24 (84.6)	61 (81.0)
	Chest pain	170 (74.2)	463 (78.3)	11 (73.3)	14 (66.7)	156 (53.2)	787 (56.8)	17 (62.3)	34 (51.9)
	Difficulty breathing	NA	NA	NA	NA	144 (47.7)	710 (51.6)	16 (56.0)	36 (55.1)
Oropharyngeal	Hoarse voice	117 (51.1)	309 (52.3)	6 (40.0)	11 (52.4)	NA	NA	NA	NA
	Sore throat	148 (64.6)	371 (62.8)	10 (66.7)	14 (66.7)	118 (38.3)	552(39.1)	15 (59.0)	29 (46.7)
	Nasal congestion	NA	NA	NA	NA	146 (48.4)	719 (51.5)	19 (61.5)	45 (56.2)
	Runny nose	NA	NA	NA	NA	116 (35.9)	636 (48.5)	14 (57.0)	33 (51.4)
Anosmia/ageusia	Anosmia	177 (77.3)	481 (81.4)	12 (80.0)	19 (90.5)	182 (63.1)	786 (56.7)	20 (75.2)	47 (70.4)
Gastrointestinal	Skipped meals	153 (66.8)	400 (67.7)	7 (46.7)	11 (52.4)	NA	NA	NA	NA
	Abdominal pain	115 (50.2)	274 (46.4)	9 (60.0)	10 (47.6)	NA	NA	NA	NA
	Diarrhoea	126 (55.0)	275 (46.5)	7 (46.7)	11 (52.4)	137 (49.2)	611 (44.4)	17 (59.8)	39 (56.1)
	Nausea or vomiting	NA	NA	NA	NA	138 (49.4)	633 (49.8)	21 (78.2)	51 (79.4)

Data are reported by pregnancy status and further subdivided by SARS-CoV-2 test positive or suspected COVID-19 status. Data are reported as N (%) in the discovery cohort, and N surveys (survey-weight adjusted %) in the replication cohort. *Fatigue* was mapped to *tiredness/exhaustion* and *unusual muscle pain* to *pain in muscle and joints* in the replication cohort. Symptoms not ascertained or mapped in either cohort are marked as not available (NA).

Among the test positives, univariate analysis on each symptom found significant effect of pregnancy with decreased odd ratio for *skipped meals* (OR 0.5, 95% CI 0.2 to 0.9) in the hospitalized women of the discovery cohort, and increased odd ratio for *nausea or vomiting* (OR 3.3, 95% CI 1.3 to 8.8) in the ‘seen at hospital’ of the replication cohort. In addition, *delirium* was associated with decreased odd ratio (OR 0.2, 95% CI 0.1 to 0.6) in the discovery cohort. In order to investigate the syndromes leading differences between pregnant and non-pregnant conditions, multivariate logistic regression was applied. In the discovery cohort, we found lower frequency of *neurologic* symptoms (t = − 7.6) for the positive hospitalized pregnant vs. non pregnant women. In the replication cohort, the *oropharyngeal* cluster was significantly more frequently reported by pregnant vs. non-pregnant tested positive (t = + 2.3); univariate test was significant even among test positives reporting being ‘seen at a hospital’ for their illness (OR 2.1, 95% CI 1.1 to 4.1) (all age-standardized and Bonferroni corrected *p* < 5e−05 for discovery and *p* < 5e−03 for replication cohorts). Results overall indicate how questions are asked can impact symptom profiles in this population.

Univariate weighted regression also showed that pregnancy had no statistically significant effect on the severity of manifestation of SARS-CoV-2 infection, when expressed as ‘severity index’ in both cohorts (*p* > 0.001, uncorrected to test the null hypothesis). In the discovery cohort, overall duration of disease was similar for pregnant and non-pregnant women. Statistically, however, time to peak of symptom manifestation was significantly longer in pregnant (mean time = 2.8 days) than in non-pregnant (2.2 days, *p* = 5.5e−7) women, though clinically the difference may be irrelevant. In the replication cohort, pregnant women who tested positive and reported being seen at the hospital similarly reported longer durations of illness.

**Figure 3:**
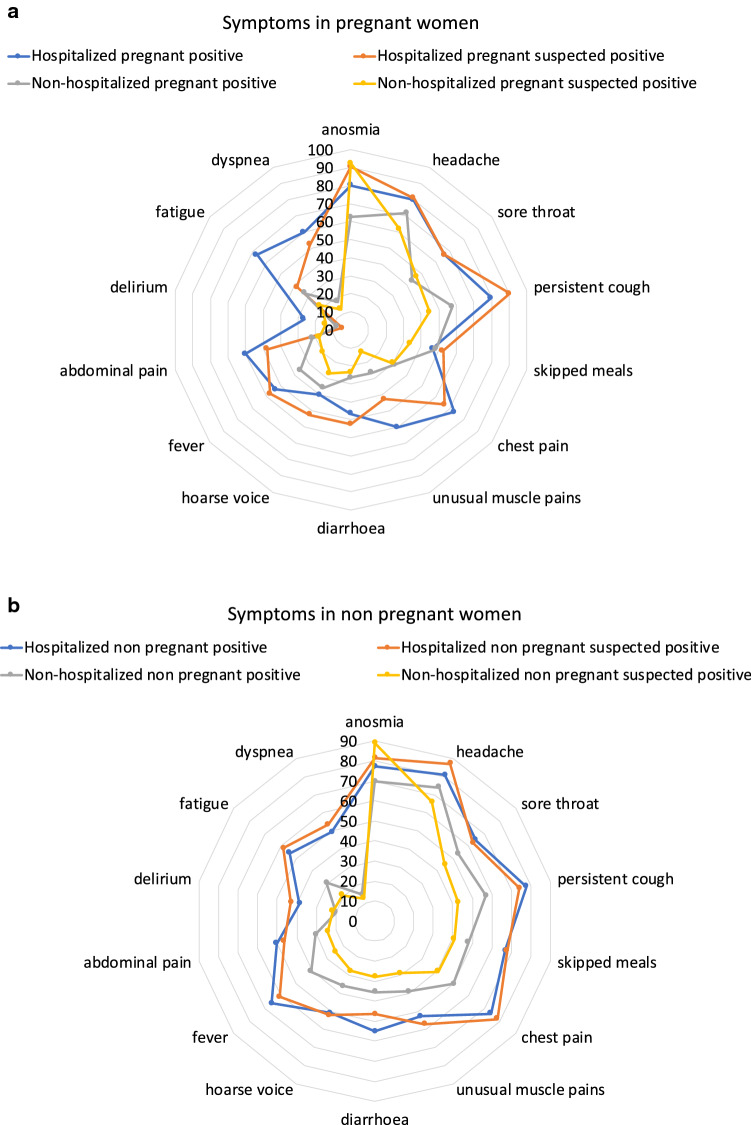
Symptom profile of hospitalized and non-hospitalized pregnant (**a**) and non-pregnant (**b**) women positive and suspected positive to SARS-CoV-2 in the discovery cohort. Results are reported in percentage of women reporting each symptom in each group.

As mentioned above, in the discovery cohort hospitalized positive pregnant women manifested persistent cough, headache and anosmia (all 80%), chest pain (73.3%), sore throat and fatigue (66.7%) as the most frequent symptoms. Non-hospitalised pregnant women positive for SARS-CoV-2 reported headache (71.9%), anosmia (62.5%), persistent cough (57.8%) and skipped meals (48.4%) most commonly (Fig. 3). See Supplementary Information S6 for the full list of symptoms and their associated prevalence.

### Comorbidities

Lung disease was the comorbidity most strongly associated with the severity of symptoms in pregnant positive women (t = 4.1 for discovery cohort; t = 14.1 for replication cohort, all *p* val < 0.0001 Bonferroni corrected).

In the replication cohort heart disease (t = 7.1) was also associated with the severity of symptoms in pregnant positive women, followed by kidney disease (t = 4.6) and diabetes (t = 3.6, all significant after Bonferroni correction at *p* val < 0.0001) (Table 3).

**Table 3:** Frequencies and percentages of comorbidities and pre-existing conditions in the discovery and replication cohorts.

Comorbidity or pre-existing condition	Discovery cohort		Replication cohort	
	Pregnant test positive (N = 79)	Pregnant suspected positive (N = 629)	Pregnant test positive (N = 134)	Pregnant suspected positive (N = 1076)
Diabetes	3 (3.8)	15 (2.4)	11 (8.9)	76 (7.4)
Lung disease	8 (10.1)	80 (12.7)	37 (31)	376 (34.2)
Heart disease	1 (1.3)	5 (0.8)	5 (6.3)	41 (4.8)
Kidney disease	0 (0.0)	2 (0.3)	8 (7.8)	30 (43.3)
Hypertension	NA	NA	17 (13.9)	170 (15.4)
Autoimmune	0 (0.0)	8 (1.3)	14 (11.5)	106 (9.3)
Cancer	0 (0.0)	1 (0.2)	5 (4.7)	29 (3.2)
Smoking Past smoker	6 (7.6) 13 (16.5)	36 (5.7) 121 (19.2)	NA	NA

Columns refer to pregnant women tested and suspected positive for SARS-CoV-2 infection. Data are reported as N (%). Data from the replication cohort are reported as N surveys (survey-weight adjusted %). Conditions not ascertained or mapped in either cohort are marked as not available (NA).

## Discussion

### Summary of the main findings

We studied two large cohorts of women, tested and suspected SARS-CoV-2 positive, with self-reported pregnancy status, symptoms and outcomes through participative surveillance. Pregnant women reported more frequent testing for SARS-CoV-2 than non-pregnant women, but generally did not experience more severe symptom profiles. Disease trajectories were similar, and the time from onset to peak of symptoms was only slightly longer in pregnant than non-pregnant women (2.8 vs. 2.2 days).

Gastrointestinal symptoms were different in pregnant and non-pregnant women with poor outcomes, with decreased *skipped meals* in the discovery cohort and increased *nausea or vomiting* in the replication cohort. Neurologic symptoms (only surveyed in the discovery cohort) were decreased in pregnant women.

The current epidemiologic literature is largely based on pregnant women admitted to the hospital, which provides a narrow view of the spectrum of SARS-CoV-2 infection in all pregnant women. Our data shows that the preponderance of tested positive and even suspected positive pregnant women were not seen at or admitted to the hospital for their illness; most pregnant women reported their recovery in the community, as was observed by Lokken et al.^20^. Cardiopulmonary symptoms were more frequently reported by pregnant women who were hospitalised. Notably, pre-existing lung disease was confirmed to be the most relevant risk factor to develop more severe COVID-19 symptoms in pregnancy, as it is outside of pregnancy. Heart disease, kidney disease and diabetes were also risk factors.

### Interpretation

Pregnant women are considered a high-risk group in UK and were considered high risk in the USA early in the pandemic. This likely contributed to the higher testing proportion but lower positive results among pregnant vs. non-pregnant women.

A recent systematic review of studies, including around 10,000 pregnant women mainly examined in hospital settings, estimated that the most common symptoms associated with the presentation of COVID‐19 were fever (75.5%, vs. 74% in non-pregnant), cough (48.5% vs. 53.5%), and myalgia (26.5% vs. 19.5%). Anosmia was found in only 13.5% of pregnant COVID‐19 patients and 25% of non‐pregnant COVID-19 women, and this could be due to the relative late discovery of anosmia as hallmark of the disease^21^. Nausea and vomiting were more frequent in pregnant patients (11% vs. 4%).

In our study, hospitalized pregnant women presented with lower frequency of neurologic symptoms compared with hospitalized non-pregnant, especially *delirium*, which was only measured in the discovery cohort. The replication cohort showed higher frequency of *gastrointestinal* symptoms among pregnant women with more severe outcomes, especially *nausea or vomiting*, which may be a feature of pregnancy itself (e.g. hyperemesis gravidum). Diarrhoea in positive pregnant women has been previously reported (rates between 8.8 and 14%)^22,23^.

Despite previous work on smaller cohorts found increased risk for development of severe COVID-19 among pregnant women compared with similarly aged adults^24,25^, syndrome severity did not differ between pregnant and non-pregnant women in both datasets of this study. This posits an equivalent manifestation of SARS-CoV-2 infection in pregnant and non-pregnant, as already reported by Chen and others^9,12^.

Pre-existing lung disease is the comorbidity with strongest impact on the SARS-CoV-2 infection severity in pregnant women in both cohorts. Lokken et al.^20^ similarly reported asthma as a primary risk factor for severe COVID-19 in pregnancy. Heart disease, kidney disease and diabetes were also associated with severity in the replication cohort, which had high enough prevalence of these conditions (related to survey-sampling to the general population) to detect an effect. These comorbidities are consistent with risk factors in the general, non-pregnant population; Li et al. observed chronic obstructive pulmonary disease, diabetes, hypertension, coronary heart disease and cerebrovascular diseases had the highest odd ratios for SARS-CoV-2 and admission to the intensive care unit (ICU)^26^, while Kumar et al. found diabetes increased SARS-CoV-2 severity and mortality two-fold^27^.

Cough, chest pain and dyspnea showed much higher incidence in the hospitalized vs. non-hospitalized pregnant women, indicating that cardiopulmonary symptoms are the major discriminant for hospitalization, and thus supporting the results reported in the systematic review by Jafari et al.^28^ Similarly, Ellington et al.^29^, found increased ICU admissions and need of mechanical ventilation in pregnant women, although the cohort studied had higher frequency of underlying medical conditions, and might be less representative of the general pregnant population.

Pregnant women with pre-existing lung disease or prominent cardiopulmonary symptoms may need special attention during the COVID-19 pandemic; lung disease had strongest impact on syndrome severity while cardiopulmonary symptoms were the main factor predicting hospitalization in pregnancy. Indeed, in pregnancy, cardiopulmonary reserve is limited which increases morbidity and complicates management when there are added physiologic stressors (e.g. asthma exacerbation)^30–33^.

Diabetes was more common in the pregnant women in our cohorts, likely of gestational origin. Consistently, Jafari et al.^28^ observed that diabetes was the most frequent comorbidity for pregnant women with COVID‐19 (18% vs. 11% non‐pregnant); specifically, non‐gestational diabetes and gestational diabetes were 8% and 10% respectively among pregnant. We also confirmed diabetes is associated to increased severity of SARS-CoV-2 symptoms^34^.

This study leveraged two cohorts followed through remote, participatory epidemiology, enabling rapid assessment of COVID-19 in pregnancy. The longitudinal nature of the discovery dataset enabled the comparison of disease duration, time from onset to peak of symptoms, and hospitalization between pregnant and non-pregnant women, prospectively. Broadly, pregnancy does not substantially contribute to morbidity in our community-based cohorts. Clinicians should be more vigilant with pregnant who have pre-existing health conditions, prominent respiratory symptoms or a higher severity index – as is the case in the general population. Further studies specifically targeting high-risk pregnancies and outcomes across the three trimesters may be warranted, to better define outcomes in this population. Also, we point out the need to interpret hospitalization rates and severity results in light of the policy changes, which can be dependent on the context or country. Last, the higher rate of caesarean deliveries observed in SARS-CoV-2 infected woman deserves to be further investigated, despite being out of the scope of this research^35–37^.

### Strengths and limitations

Participatory surveillance tools are crucial to epidemiological research and citizen science, as they increase population’s awareness of urgent public health risks, promote public participation into science and enable inclusion in studies of large samples from the community within short time periods. Real-time public health data has been crucial in decision-making during the COVID-19 pandemic. However, user of smartphone applications and web-based surveys may be not representative of the general population, potentially limiting generalizability of the findings. Self-reported events may suffer from misclassification bias, which may be differential (e.g. ability to log hospitalization may be higher in less severely affected participants, test results known at the time of cross-sectional symptom reporting may differ). Median app usage was 18 days, which may be insufficient follow-up to ascertain all outcomes. In the discovery cohort, pregnancy status was only queried at the time of registration; women who became pregnant after registration may be misclassified. In addition, gestational age during the infection could not be assessed, as well as whether women were symptomatic at the time of delivery. The replication cohort was designed to be representative of USA population through survey sampling for the active user base and weights with raking to the USA census. Despite the different platforms and countries of origin of users, the cross-sectional surveys showed similar results to the detailed longitudinal discovery cohort of technology-aware smartphone users. However, it was not possible to distinguish difference in methodology from country-specific effects. Additionally, we applied age-standardization to account for demographic structure inherent to pregnancy. Despite the differences in the UK, USA and Sweden testing guidelines and healthcare systems, COVID-19 morbidity in pregnancy was comparable. We were able to develop and validate a prediction score for suspected positive, as well as a severity score for use in women of childbearing age; and these performed similarly in the cross-sectional survey data despite development using longitudinal symptom reports. This may be useful for obstetricians in the context of limited access to SARS-CoV-2 testing during this pandemic.

## Conclusions

Our findings from two large real-time syndromic surveillance technologies provide evidence that most pregnant women in the community who are positive for SARS-CoV-2 are at similar risk of developing either increased morbidity or complex symptomatology compared with non-pregnant women. However, pre-existing lung or cardiac disease may exacerbate cardiopulmonary stress of pregnancy. Pregnant women with comorbidities appear to be at increased risk for severe disease, consistent with evidence from COVID-19 infection in the general population. Pregnant women with pre-existing conditions, similar to the general adult population, require careful monitoring for the evolution of their symptoms during SARS-CoV-2 infection.

## Materials and methods

### Study populations

#### Discovery cohort

The COVID Symptom Study smartphone-based application (app), developed by Zoe Global Limited, and having almost four million users from the general population in UK, 280,000 from USA and around 175,000 from Sweden. Recruitment has been conducted through variegated advertisement campaigns (web-site, newspapers, etc.). Users self-report daily information about their overall health status, as well as their symptoms (from a pre-defined list, to standardise input)^21,38^. We included all pre-menopausal (if menopausal status was reported) women aged 18 to 44 years, who used the app between 24 March and 7 June 2020, and specified their pregnancy status at baseline (pregnant or not pregnant), including symptom profiles, outcomes on testing positive for SARS-CoV-2, and hospitalization (Supplementary Information S1).

#### Replication cohort

The COVID-19 Symptom survey, hosted by the Delphi Group at Carnegie Mellon University and distributed with the support of Facebook. Surveys were conducted using sampling strategies and survey weights to ensure respondents were representative of the USA source population^39^ (Supplementary Information S1). Using data from launch (6 April 2020) through 7 June 2020, we identified surveys from 1,344,966 female respondents who indicated their pregnancy status and age 18–44 years^40^. Users specified if they had experienced specific symptoms over the last 24 h, in addition to answering demographic and infection-related questions.

Participation of individuals from both cohorts was voluntary, and informed consent was obtained to use personal data in aggregation for research purposes. All methods were performed in accordance with the relevant guidelines and regulations, thus including the Declaration of Helsinki and subsequent updates. Details on ethical permissions are reported at the end of this manuscript.

### Pregnancy groups, symptoms, syndromes and outcomes

#### Pregnancy status

Women were divided into pregnant and non-pregnant subgroups, based on self-reported pregnancy status, ascertained once near the start of follow-up in the discovery cohort, and at each survey for the replication cohort. Gestational age, at the time pregnancy was ascertained, was available only for the discovery cohort.

#### COVID-19 test and suspected positive

Self-reported COVID-19 testing was used to identify women with SARS-CoV-2 infection (termed *test positive*). Test positives were considered *symptomatic positive* if they reported at least one of the tracked symptoms. The type of test (e.g. PCR, serology) was not ascertained, and those reporting a pending test were excluded.

Suspected positive cases were imputed, based on a previously published method for the computation of a test-positive prediction score^21^. The model was retrained for pregnancy age distribution, based on a bootstrapped train-test scheme in the discovery cohort, and using a strict mapping to equate symptoms ascertained in both the discovery and replication cohorts. We defined the outcome of suspected COVID-19 (termed *suspected positive*) for anyone with a score-based imputation probability above a computed threshold (Supplementary Information S2).

#### Hospitalization and syndrome severity

Individuals were considered to have been hospitalized when they indicated being either admitted to or discharged from hospital in their daily reporting, within one week before/after reporting at least one of the tracked symptoms. Symptoms, test results and hospitalization can be reported anytime and with no interdependencies in the app, and symptom reporting is not censored after input of test results. Symptom severity was thus defined as the weighted sum of symptoms based on peak presentation when comparing individuals reporting hospital visit with individuals who did not, in the training set of the discovery cohort (Supplementary Information S3). Symptoms were equated in the two cohorts.

The weighting was then normalized so that the severity index ranges from 0 (no symptom) to 1 (all symptoms).

### Statistical analysis

A power analysis was conducted to assess the suitability of the samples size. To account for the difference in age distributions between pregnant and non-pregnant groups, age-standardization was performed, by calculating weights for the non-pregnant women, to standardize to the age-distribution of the pregnant population (Supplementary Information S4 and S5).

#### Symptoms

To explore differences in the symptom profile between pregnant and non-pregnant women who tested or were suspected positive for SARS-CoV-2 and who also required hospitalization or sought care, we applied univariate unconditional age-weighted logistic regression for each of 18 symptoms ascertained in either the discovery cohort, the replication or in both. We then conducted multivariate analysis on symptoms grouped into clusters by body system, as shown in Table 2, and normalized to range from 0 to 1.

#### Severity of syndrome

To assess symptom severity differences between pregnant and non-pregnant women who tested or were suspected positive for SARS-CoV-2 infection and were hospitalized, univariate unconditional age-weighted regression was applied to the pregnant and non-pregnant groups of the discovery cohort, with the severity index as a response variable. The analysis was repeated for the replication cohort among those who reported to have been ‘seen at a hospital for their symptoms’, conditional on those who predicted or tested positive for SARS-CoV-2.

#### Hospitalization

To explore differences in the symptom profiles between hospitalized and non-hospitalized pregnant women positive for SARS-CoV-2, the frequency and percentage of women reporting each symptom were calculated for the discovery cohort. Symptoms were ranked from the most to the least frequently reported.

#### Disease modifiers

To identify demographic characteristics, comorbidities and pre-conditions associated with COVID-19 symptom severity in pregnancy, a multivariate unconditional regression was applied to each dataset, with the severity index as a response variable and age, diabetes, heart, lung (and asthma) and kidney diseases as factors. As the regression investigated within-group factors, age-weighting was not applied. Bonferroni correction for multiple tests was applied.

Statistical analyses were performed using STATA version 16 (discovery cohort) and R 3.6.3 (replication cohort).

### Ethics approval

The study involving the discovery cohort has been approved by KCL Ethics Committee REMAS ID 18210, review reference LRS-19/20-18210 and all participants provided consent. Data from the replication cohort were acquired under the approval from the Boston Children Hospital’s Institutional Review Board, review reference BCH IRB-P00023700.

## Supplementary Information

Supplementary Information.

## Data Availability

Data collected in the COVID-19 Symptom Study smartphone application are being shared with other health researchers through the UK National Health Service-funded Health Data Research UK (HDRUK) and Secure Anonymised Information Linkage consortium, housed in the UK Secure Research Platform (Swansea, UK). Anonymised data are available to be shared with HDRUK researchers according to their protocols in the public interest (https://web.www.healthdatagateway.org/dataset/fddcb382-3051-4394-8436-b92295f14259). US investigators are encouraged to coordinate data requests through the Coronavirus Pandemic Epidemiology Consortium (https://www.monganinstitute.org/cope-consortium).
