# Supplementary material for: Symptoms and syndromes associated with SARS-CoV-2 infection and severity in pregnant women from two community cohorts
Source: Sci Rep. 2021 Mar 25;11:6928. doi: 10.1038/s41598-021-86452-3 (PMC7994587; doi:10.1038/s41598-021-86452-3)
Supplement: Supplementary file 1 — Supplementary Information. [file 41598_2021_86452_MOESM1_ESM.pdf]

SUPPLEMENTARY INFORMATION

Symptoms and syndromes associated with SARS-CoV-2 infection and severity in pregnant women from two community cohorts

Erika Molteni and Christina M. Astley and Wenjie Ma, Carole H Sudre, Laura A. Magee, Benjamin Murray, Tove Fall, Maria F. Gomez, Neli Tsereteli, Paul W. Franks, John S. Brownstein, Richard Davies, Jonathan Wolf, Tim D Spector, Sebastien Ourselin, Claire J Steves, Andrew T Chan and Marc Modat.

Supplementary Material S1. Study populations

Discovery Cohort. The COVID Symptom Study smartphone-based application (app) was launched in the United Kingdom (UK) on 24 March 2020 and in the United States of America (USA) and Sweden on 29 March 2020. It was developed by Zoe Global Limited, with input from clinicians and scientists from King's College London and Massachusetts General Hospital. After three months of activity, data were available for more than four million users from the general population in UK, Sweden and USA. The aim of the app was to enable users to self-report information about their overall health status, as well as a set of pre-specified symptoms [1]. The survey questions have been published previously [2]. Self-reported location, age and core health risk factors are recorded at first use. Daily updates on symptoms, SARS-CoV-2 testing results and healthcare received are encouraged through daily notifications. Updates from asymptomatic individuals are also encouraged. Data from a cohort

of pre-menopausal (if menopausal status was reported) women aged 18 to 44 years (~1,3 millions) was extracted on 7 June 2020. Data from women who specified their pregnancy status at baseline (pregnant or not pregnant) was selected (N= 1,170,315). All data available at that date were used for further analysis, and one third was used as a test set (N=400,750, see next section). Symptom profiles, and outcomes on testing positive for SARS-CoV-2, including hospitalization, were ascertained prospectively.

Replication Cohort. The Facebook COVID 19 Symptom survey was launched in the USA on 6 April 2020 (hosted by the Carnegie Mellon Delphi Research Center) and internationally later in April 2020 (hosted by University of Maryland Joint Program on Survey Methodology). By 7 June 2020, there were 1,876,130 female respondents, of whom 1.344.966 who indicated their pregnancy status (pregnant or not pregnant) and age 18-44 years [3]. Each day, a random sample, designed to achieve representative surveys of the Facebook Active Users Base (FAUB) was selected to be surveyed using the Total Survey Error Framework. The USA FAUB is approximately 253 million of the 328 million USA population. Users are presented a cross-sectional survey where they specify if they have experienced specific symptoms over the last 24 hours, in addition to answering demographic and infection-related questions. A non-reversible unique identifier associated with each survey response is used to generate a survey weight, calculated as the inverse probability score weighting of non-response and covariates, including age, gender, geographical variables and other attributes that correlate with survey outcomes. Post stratification raking is then applied based on age, gender and state using USA census and United Nations Population Divisions. Survey weights are used to create a weighted sample to improve representation of the source population. Details of the survey questionnaire are available online [4]. This sampling procedure was designed to achieve representative samples of the Facebook active user base. Weighting of survey responses accounts for nonresponse in the user base and

raking to the US census data (age, gender, geographic location). Due to the size of the US Facebook active user base from which surveys were sampled, it can be expected that data from each survey correspond to distinct pregnancies; however, due to the anonymity of the data, repeated survey participation cannot be excluded.

Supplementary Material S2. Training, validation, test and replication of the regression method for the test-positive prediction score imputation. All non-pregnant women aged 18-44 in the discovery cohort with a definite SARS-CoV-2 test result were split into 3 sets (training, validation, test) with similar distributions of test results (positive/negative) within 1 week before or after peak sum of symptoms. The training set was further subset to ensure the age distribution of those with positive and negative tests was comparable to the pregnant population aged 18-44 years in the discovery cohort. The multivariable logistic regression with all symptoms at the time of peak symptoms plus age as covariates and test result as the outcome was then trained by bootstrapping 100 random draws from the training set. The coefficients of the final model were as follows:

Variable	Mean	SD
Intercept	-2.072137	0. 140457
Abdominal pain	-0.344712	0. 087838
Age	-0.011956	0. 003853
Chest pain	0.195357	0. 061862
Delirium	-0.139931	0. 087323
Diarrhoea	0.061036	0. 075804
Severe fatigue	-0.003907	0. 102081
Fever	0.019550	0. 065003
Headache	0.141982	0. 066226

Hoarse Voice	-0.032844	0. 068308
Loss of smell	2.378318	0. 057300
Persistent Cough	0. 495585	0. 063387
Skipped Meals	0. 2054785	0. 064817
Shortness of Breath (dyspnea)	0.287359	0. 130670
Sore Throat	-0.478740	0. 063876
Unusual Muscle pains	0.363984	0. 067883

On the training set, a threshold of th = 0.170365, corresponding to the Youden index, was adopted for the binarization of the imputation of SARS-CoV-2 test status (suspected positive). Measures of central tendency and variation of the coefficients were then used to assess performance on the validation set. These coefficients were then applied to impute in the test set of non-pregnant women plus the pregnant population. Receiving Operative Curves testing the suspected positive imputation model in the non-pregnant validation, non-pregnant test, and pregnant test sub-cohorts (restricted to those with a positive or negative SARS-CoV-2 test) are shown below.

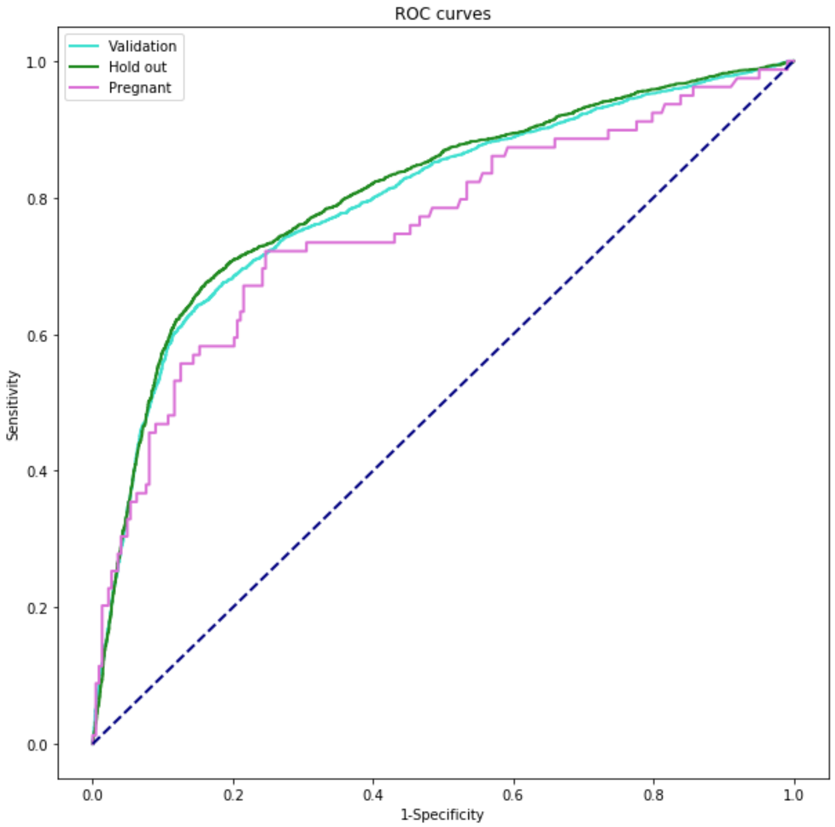

To validate the above symptom score for imputation of suspected positives in the replication cohort, and to conduct a sensitivity analysis of methods for equalizing the scores in the two cohorts, we compared three methods for mapping symptoms from those ascertained in the discovery to those ascertained in the replication cohort. The first method (relaxed mapping) mapped all discovery symptoms to the nearest replication

symptom, substituting synonyms of medical term and then symptoms from the same organ system until all discovery symptoms had a single proxy symptom in the replication set. The second method (moderate mapping) allowed for synonym symptoms but only a limited range of organ system symptom substitutions. The third method (stringent mapping) only allowed for limited synonym symptoms and no organ system symptom substitutions. For the latter two scores, unmapped symptoms were set to false or 0 (symptom not present) in the replication score calculation. All scores were normalized to range from 0 (no symptoms) to 1 (all symptoms). Below is a table comparing the three symptom mapping methods:

Replication variables mapped to Discovery variables	Stringent	Moderate	Relaxed
Anosmia	smelltaste	smelltaste	smelltaste
Delirium	0	0	tiredexh
Headache	0	0	tiredexh
Fatigue	tiredexh	tiredexh	tiredexh
Unusual muscle pains	muscjoint	muscjoint	muscjoint
Fever	fever	fever	fever
Persistent_cough	cough	cough	cough
Shortness of breath	sob	sob	sob
Chest pain	presspainchest	presspainchest	presspainchest
Sore throat	sorethroat	sorethroat	sorethroat
Hoarse voice	0	sorethroat	sorethroat
Skipped meals	0	nauseavomit	nauseavomit
Abdominal pain	0	0	nauseavomit or diarrhoea
Diarrhoea	diarrhoea	diarrhoea	diarrhoea
Age (1 year increments)	age (10 year categories)	age (10 year categories)	age (10 year categories)

The Area Under the Curve for the ROC for each mapping scheme (stringent, moderate, relaxed) assessed among the pregnant (P) and non-pregnant (N) populations from the Replication Cohort (All) are shown below. Stringent mapping had the highest AUC the pregnant population, while there were substantially more non-pregnant women in the cohort.

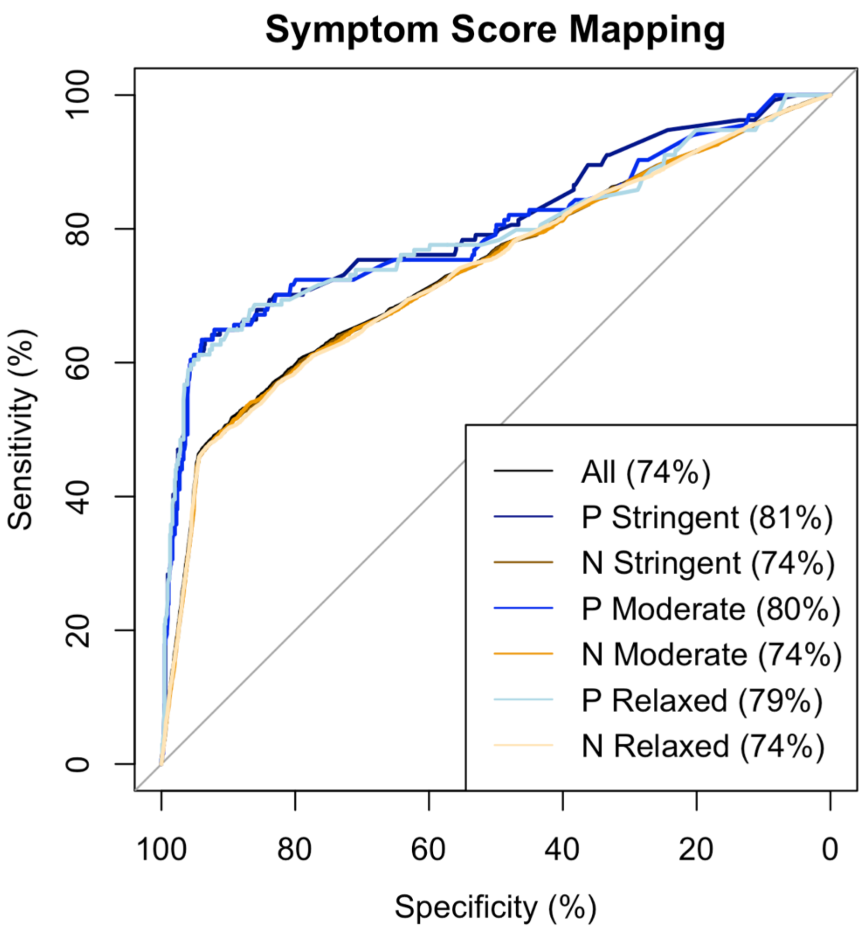

To better understand the effect of age, we further explored stringent score performance in each of the replication cohort age categories, in 10 year increments, for the pregnant (P20, P30, P40) and non-pregnant (N20, N30, N40). Score performance was the best among the 25 to 34 year olds, which is reflective of the largest age category in the discovery cohort data on which the score was trained (as it was standardized to the pregnant women age distribution, though in 1 year rather than 10 year age bins).

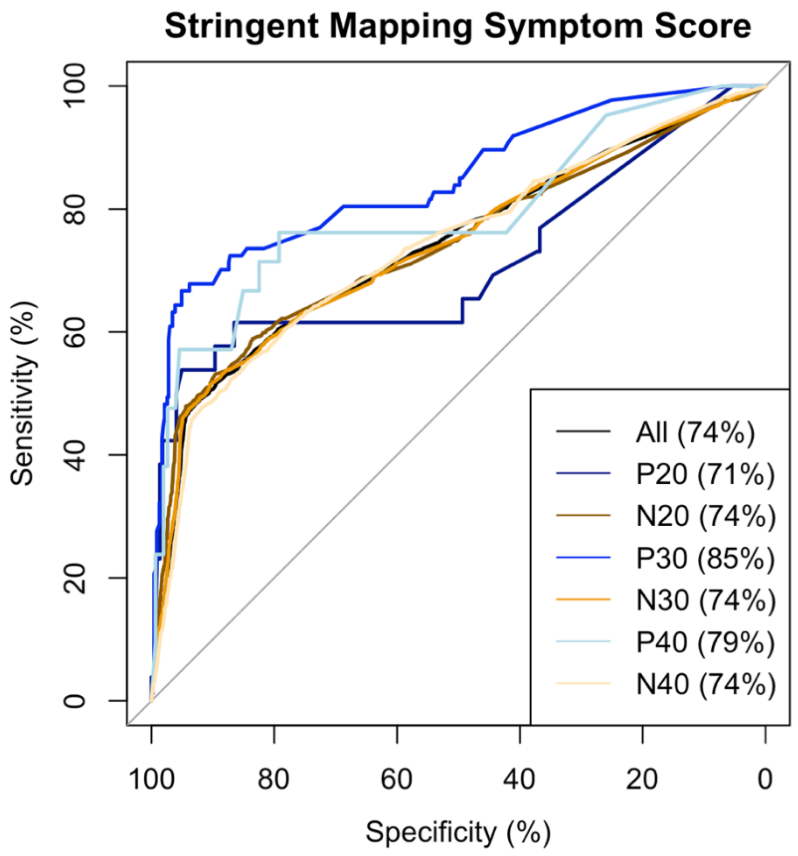

Supplementary Material S3. Severity index calculation. Symptom severity is defined as the weighted sum of symptoms based on hospital presentation. For each symptom, the weights are calculated as the ratio between the frequency of occurrence in the hospitalised population compared to the non-hospitalised population in the training set of positive non-pregnant women, age 18-44 years, in the discovery cohort. This score is normalized to range from 0 and 1. In the replication cohort, stringent mapping of symptoms (see above) was used to equalize symptoms for the severity score, using the same weights developed in the discovery cohort. The severity score was then rescaled to be between 0 and 1, based on the maximum possible severity with the mapped symptoms.

Severity = 0.063 abdominal_pain + 0.063 chest_pain + 0.046 sore_throat + 0.200 dyspnea + 0.093 fatigue + 0.043 headache + 0.051 hoarse_voice + 0.043 anosmia + 0.087 delirium + 0.063 diarrhoea + 0.059 fever + 0.055 persistent_cough + 0.064 unusual_muscle_pains + 0.068 skipped_meals

Supplementary Material S4.

Power Analysis. We conducted a power analysis for multiple regression with the syndrome severity as a response variable, pregnancy condition as fixed factor, and effect equal to the difference of R^2 of the full equation and the one reduced for the target factor. We employed *powerreg* command in STATA v.16, with statistical power set at s=0.90. The results showed that we were sufficiently powered to investigate the overall syndrome severity and the grouped symptoms, but not anosmia alone. Sample sizes are reported in the table below:

GROUP of SYMPTOMS	Number of subjects
All symptoms	21
All cluster of symptoms (body systems)	17
Inflammation	41
Neurologic	63
Cardiopulmonary	39
Oropharyngeal	117
Gastrointestinal	36
Anosmia	6720

Age Standardization. To account for the difference in age distributions between pregnant and non-pregnant groups, the non-pregnant women were weighted to standardize to the age-distribution of the pregnant population (Supplementary Material 5). To calculate the ratio of tested women in the pregnant and non-pregnant group, we computed the ratio between the sum of weights of the tested women and the sum of weights of all women in the same group. In the replication cohort, survey-specific weights were applied in addition to age-standardization to the pregnant population.

$$\hat{r} = \frac{\sum_{j \in S} v_j w_j y_j}{\sum_{j \in S} v_j w_j z_j} \tag{1}$$

Where $\hat{r}$ is the ratio, v_j are the survey weights, w_j are the age weights, y_j is test availability, z_j indicates all the women in the group, and j indicates the single subjects. Analogously, variance estimates were calculated, accounting for age standardization and survey-specific weights:

$$\hat{V} = \frac{\sum_{j \in S} x_j^2 \left(y_j - \hat{r} z_j\right)^2}{\left(\sum_{j \in S} x_j z_j\right)^2} \tag{2}$$

with $x_j=v_jw_j$.

Total reports are raw counts. Means and ratios are adjusted for survey weights and age-standardization. Ratios of tested women in the pregnant and non-pregnant groups were computed as described above.

Assessment of social status comparability. To rule out any confounding effect of the women's social status on our analyses, we compared the Index of Multiple Deprivation (IMD) in the pregnant and non-pregnant groups, available from the discovery cohort. Median IMD was 6 for both groups, and rank sum test excluded any significant difference between the two groups (Z=0.82, p-val=0.4125).

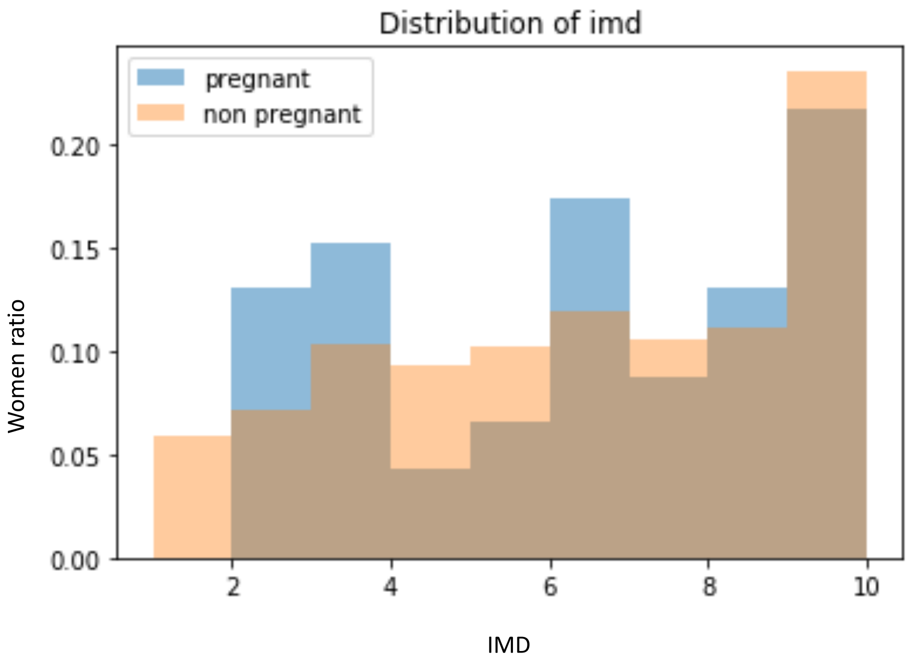

Supplementary Material S5. To account for the difference in demographic distributions between the pregnant and non-pregnant groups in each dataset, age standardisation was performed. A weight equal to 1 was assigned to every woman in the pregnant group. Women in the pregnant and non-pregnant groups were assigned to one-year age ranges, and frequencies were obtained for each age range and for each group. Histogram normalisation of the age distribution of the non-pregnant women was then calculated as the inverse probability to match the age distribution of the pregnant women considering separately the positive and the imputed group. Overall, this was calculated as the ratio between the frequencies in the specific age range of the two normalised histograms of age. For instance, weights assigned to non-pregnant women aged above 40 are smaller than 1, to compensate for the natural decrease of frequency of pregnancies after the age of 40 years.

Supplementary Material S6. Symptomatic profile of hospitalized and non-hospitalized pregnant women positive and suspected positive to SARS-CoV-2. Symptoms are ranked from the most to the least frequent. Results are reported in percentage of women reporting each symptom in each group.

Hospitalized		Non-hospitalized	
Pregnant positive	Pregnant suspected positive	Pregnant positive	Pregnant suspected positive
80.0 Persistent cough	90.5 Persistent cough	71.9 Headache	92.4 Anosmia
80.0 Headache	90.5 Anosmia	62.5 Anosmia	62.0 Headache
80.0 Anosmia	81.0 Headache	57.8 Persistent cough	46.9 Sore throat
73.3 Chest pain	66.7 Chest pain	48.4 Skipped meals	44.7 Persistent cough
66.7 Sore throat	66.7 Sore throat	43.8 Sore throat	33.7 Skipped meals
66.7 Fatigue	57.1 Fever	35.9 Fever	29.8 Chest pain
60.0 Dyspnea	52.4 Dyspnea	35.9 Hoarse voice	26.8 Hoarse voice

60.0 Unusual muscle pain	52.4 Skipped meals	32.8 Fatigue	23.7 Diarrhoea
60.0 Abdominal pain	52.4 Diarrhoea	31.3 Chest pain	21.7 Fatigue
53.3 Fever	52.4 Hoarse voice	26.6 Diarrhoea	19.9 Fever
46.7 Skipped meals	47.6 Abdominal pain	26.6 Unusual muscle pain	18.1 Abdominal pain
46.7 Diarrhoea	42.9 Unusual muscle pain	21.9 Abdominal pain	14.3 Delirium
40.0Hoarse voice	38.1 Fatigue	17.2 Dyspnea	13.7 Unusual muscle pain
26.7 Delirium	4.8 Delirium	7.8 Delirium	12.7 Dyspnea

References

1. Drew DA, Nguyen LH, Steves CJ, Menni C, Freydin M, et al. Rapid implementation of mobile technology for real-time epidemiology of COVID-19. Science (80-). 2020;
2. Menni C, Valdes AM, Freidin MB, Sudre CH, Nguyen LH, Drew DA, et al. Real-time tracking of self-reported symptoms to predict potential COVID-19. Nat Med. 2020;
3. Kreuter F, Barkay N, Bilinski A, Bradford A, Chiu S, Eliat R, et al. Partnering with Facebook on a university-based rapid turn-around global survey. Surv Res Methods. 2020;14(2).
4. Facebook Questionnaire [Internet]. p. https://cmu.ca1.qualtrics.com/jfe/preview/SV_cT2ri. Available from: https://cmu.ca1.qualtrics.com/jfe/preview/SV_cT2ri3tFp2dhJGZ?Q_SurveyVersionID=current&Q_CHL=preview
